# Surfactant protein-A nanobody-conjugated liposomes loaded with methylprednisolone increase lung-targeting specificity and therapeutic effect for acute lung injury

**DOI:** 10.1080/10717544.2017.1402217

**Published:** 2017-11-21

**Authors:** Nan Li, Dong Weng, Shan-Mei Wang, Yuan Zhang, Shan-Shan Chen, Zhao-Fang Yin, Jiali Zhai, Judy Scoble, Charlotte C. Williams, Tao Chen, Hui Qiu, Qin Wu, Meng-Meng Zhao, Li-Qin Lu, Xavier Mulet, Hui-Ping Li

**Affiliations:** a Department of Respiratory Medicine, Shanghai Pulmonary Hospital, Tongji University School of Medicine, Shanghai, China;; b Department of Respiratory Medicine, People’s Hospital Affiliated to ZhengZhou University, ZhengZhou, China;; c School of Medicine, Suzhou University, SuZhou, China;; d CSIRO Manufacturing, Clayton, Australia;; e CSIRO Manufacturing, Parkville, Australia

**Keywords:** Acute lung injury, bioconjugation, liposomes**;** lung-targeted drug delivery, surfactant protein-A

## Abstract

The advent of nanomedicine requires novel delivery vehicles to actively target their site of action. Here, we demonstrate the development of lung-targeting drug-loaded liposomes and their efficacy, specificity and safety. Our study focuses on glucocorticoids methylprednisolone (MPS), a commonly used drug to treat lung injuries. The steroidal molecule was loaded into functionalized nano-sterically stabilized unilamellar liposomes (NSSLs). Targeting functionality was performed through conjugation of surfactant protein A (SPANb) nanobodies to form MPS–NSSLs–SPANb. MPS–NSSLs–SPANb exhibited good size distribution, morphology, and encapsulation efficiency. Animal experiments demonstrated the high specificity of MPS–NSSLs–SPANb to the lung. Treatment with MPS–NSSLs–SPANb reduced the levels of TNF-α, IL-8, and TGF-β1 in rat bronchoalveolar lavage fluid and the expression of NK-κB in the lung tissues, thereby alleviating lung injuries and increasing rat survival. The nanobody functionalized nanoparticles demonstrate superior performance to treat lung injury when compared to that of antibody functionalized systems.

## Introduction

Glucocorticoids (GCs) have strong anti-inflammatory and immunosuppressive effects, and thus are widely used to treat inflammatory and autoimmune diseases (Gajic et al., 2011). Aerosol inhalation of GCs to the lung is in general considered to be safe and usually effective for inflammatory airway diseases such as COPD and asthma. However, since aerosol GCs mainly deposit in the airway and hardly reach the alveoli, the bioavailability of GCs to lung parenchyma and interstitial tissue is actually very low (Pan et al., 2009; Zhai et al., 2015). Thus, aerosol delivery is not effective for distal lung parenchyma and interstitial lung diseases. Intravenous administration of drug is usually required to treat parenchymal and interstitial lung diseases.

Acute lung injury (ALI) and its advanced-stage acute respiratory distress syndrome (ARDS) (Ferguson et al., 2004; Angus, 2012; Ranieri et al., 2012; Thompson & Matthay, 2013) cause extremely high mortality, and thus are considered as the most serious among all respiratory system disorders. ALI/ARDS damages the alveolar epithelial cells and capillary endothelial cells through various direct or indirect mechanisms, resulting in diffuse pulmonary interstitial and alveolar edema (Greenstein et al., 2002; Curtis et al., 2006). The pathophysiological basis is the inflammation cascade involving a variety of inflammatory cells, mediators, and cytokines (Warrington & Bostwick, 2006; Angus, 2012). As one of the most important drugs for ALI/ARDS treatment, methylprednisolone (MPS) can effectively control early inflammation, improve respiratory function, and inhibit pulmonary fibrosis (Irwin & Richardson, 2006; Gajic et al., 2011). They have also been used in the long-term treatment of many other pulmonary disorders, including various types of interstitial lung diseases, sarcoidosis, etc. (Rochat & Janssens, 2012). Even though GCs are very effective for pulmonary inflammation, GCs have a series of adverse effects, such as aggravating infections, increasing blood pressure and blood sugar, and causing peptic ulcers, gastrointestinal bleeding, Cushing syndrome, osteoporosis, aseptic necrosis of bones, fractures, delayed wound healing, growth retardation in children, and even more severe adverse effects that are potentially crippling or life threatening (Courrier et al., 2002; Dandekar et al., 2010). The distribution of GC hormone in the body is not specific and selective, high-dose GC is usually required to achieve satisfactory therapeutic efficacy in the diseased organ, which consequently and unavoidably causes side effects. A reduction of these adverse side effects is of clear importance in the treatment of lung disease and injury. Organ-targeting drug-delivery will reduce systemic exposure to GC. Herein, we structure and demonstrate the *in vivo* efficacy of novel lung-targeting liposomal particles.

The moiety chosen to permit efficacious targeting to the lung is surfactant protein A (SP-A) as it shows the most abundant expression in type II alveolar epithelial cells while it is rarely expressed in extrapulmonary tissues or organs (Schmiedl et al., 2005). Our previous study exploited SP-A polyclonal antibodies as lung-specific targeting molecules conjugated to dexamethasone loaded liposomes to form active nanoparticles, and *in vivo* experiments confirmed the feasibility of using SP-A as a lung-specific targeting agent (Chen et al., 2013). Rapid clearance from circulation of the antibody was observed, which is potentially associated with the high molecular weight of the complete antibody. The performance of the antibody was not very satisfactory because of low tissue penetration and strong immunogenicity (Chen et al., 2013). To overcome these defects, we subsequently developed an anti-rat SP-A nanobody with low molecular weight, effective tissue penetration, high affinity, stable structure, and low immunogenicity following previous methods (Hamers-Casterman et al., 1993; Siontorou, 2013; Desmyter et al., 2015), and proved that these anti-rat SP-A nanobodies display higher safety and more specific lung-targeting properties compared with SP-A polyclonal antibodies (Wang et al., 2015).

The ultimate aim of these functional nanoparticles is not only to retain or improve the efficacy of the drug payload but also to drastically reduce adverse side effects (Bernard et al., 1994; Ashbaugh et al., 2005; Maybauer et al., 2006; Buregeya et al., 2014). The attenuation of side effects can be achieved by targeting the desired tissues or lesions. In the current study, we demonstrate the development of lung-targeting MPS-loaded liposomes and their efficacy, specificity, and safety. The amphiphilic steroidal molecule was loaded into functionalized sterically stabilized unilamellar liposomes (NSSLs). Targeting functionality was added to the liposomes by post-drug-loading conjugation of surfactant protein A nanobodies (SPANb) to form liposomal targeted particles (MPS–NSSLs–SPANb). In this study, we found that MPS–NSSLs–SPANb exhibited good size distribution, morphology and encapsulation efficiency. MPL-NSSLs-SPANb therapy targets the lung specifically, reduces lung inflammation, and decreases liver and renal toxicity. The nanobody functionalized nanoparticles demonstrate superior performance to treat lung injury in animal models when compared with antibody functionalized systems.

## Materials and methods

### Materials

1,2-Distearoyl-*sn*-glycero-3-phosphocholine (DSPC), nitrobenzofurazan (DSPE-PEG-NBD) was purchased from Avanti Polar Lipids, Inc. (Alabaster, AL). 1,2-Distearoyl-*sn*-glycero-3-phosphoethanolamine-PEG_3400_-NH_2_ (DSPE-PEG_3400_-NH_2_) was purchased from Nanocs Inc. (New York, NY). *N*-Succinimidyl iodoacetate (SIA crosslinker) was purchased from Thermo Scientific (Waltham, MA). Phosphate buffered saline (PBS, pH 7.4) and cholesterol (CHOL) were purchased from Sigma-Aldrich (St. Louis, MO). SP-A antigen was synthesized by Shanghai YouLong Biotech Co., Ltd. (Shanghai, China). SP-A nanobody (SPANb) was synthesized by the Laboratory of Respiratory Disease, Shanghai Pulmonary Hospital, China (Gajic et al., 2011). All other chemicals and solvents were of analytical grade. Milli-Q H_2_O (18.2 MΩ) was used for all aqueous preparations. All compounds were used without further purification.

### Preparation of methylprednisolone-loaded NSSLs using a remote loading method with pH gradient

#### Synthesis of DSPE-PEG_3400_-iodoacetate

To synthesize DSPE-PEG_3400_-iodoacetate, DSPE-PEG_3400_-NH_2_ was activated by SIA (Buregeya et al., 2014) at a mole ratio of 1:150 in chloroform and kept in the dark at room temperature overnight. The resulting DSPE-PEG_3400_-iodoacetate was purified by PD-10 desalting column (GE Healthcare, Otelfingen, Switzerland) in Milli-Q water to remove excess SIA. DSPE-PEG_3400_-iodoacetate was lyophilized (Scitek, Lane Cove, Australia).

#### Preparation of NSSLs exhibiting calcium acetate gradient

NSSLs were prepared by thin lipid film formation followed by hydration and extrusion through polycarbonate membranes (Ashbaugh et al., 2005). DSPC (20 mM), CHOL (14.5 mM), and DSPE-PEG_3400_-iodoacetate (1.8 mM) were dissolved in a chloroform–methanol mixture (volume ratio 2:1). A thin lipid film was prepared using N_2_ gas and placed under vacuum overnight to evaporate the organic solvents. Calcium acetate (200 mM) was added into the lipid film for hydration, and then the hydrated lipid film was sonicated in a water bath sonicator (Unisonics, Brookvale, Australia) at 60 °C for 30 min. The sonicated solution was extruded through a 0.1-µm polycarbonate filter membrane (Nuclepore Track-Etched Membrane, Whatman, Clifton, NJ) 13 times using a mini-extruder (Avanti Polar Lipids, Inc., Alabaster, AL). To create the transmembrane calcium acetate gradient, the calcium acetate of the external liposome medium was replaced by 0.9% NaCl (w/v) in four dialysis steps overnight (Bernard et al., 1994) using a dialysis tube (Float-A-Lyzer G2, Spectra Por).

#### Remote loading of MPS into NSSLs to form MPS–NSSLs

MPS was dissolved in 0.9% NaCl (w/v) and then mixed with preformed NSSLs exhibiting a transmembrane calcium gradient. The mixture of MPS and NSSLs was incubated at 65–70 °C for 1 h. Then, MPS–NSSLs were purified by gel filtration on a Superose 12 10/300 column (GE Healthcare, Chicago, IL) equilibrated with PBS.

### Conjugating SPANb to MPS–NSSLs to form MPS–NSSLs–SPANb

SPANb was mixed with MPS–NSSLs and then incubated at 4 °C overnight for conjugation. SPANb and MPS–NSSL were mixed in different mole ratios of His-tag to iodoacetate to optimize conjugation efficiency. Sodium dodecyl sulfate polyacrylamide gel electrophoresis (SDS-PAGE) analysis on an XCell SureLock Mini-Cell Electrophoresis System (Invitrogen, Carlsbad, CA) was performed to confirm the conjugation of SPANb to MPS–NSSLs. Conjugation efficiency in percentage was calculated by integrating the stained gels using Image J software (Bethesda, MD) (Gajic et al., 2011). After conjugation, MPS–NSSLs–SPANb (the mixture of SPANb and MPS-NSSLs) was purified by gel filtration on a Superose 12 10/300 column (GE Healthcare, Chicago, IL) equilibrated with PBS to remove of the free SPANb. For the preparation of FITC-labeled MPS-NSSLs-SPANb (MPS-NSSLs-SPANb-FITC), the same protocol was used by replacing SPANb with FITC labeled SPANb (SPANb–FITC). DSPE-PEG-NBD was used to replace/mixed with MPS, and then MPS-NSSLs-NBD/NSSLs-NBD was prepared using the same method.

### Binding assay of MPS–NSSLs–SPANb

An ELISA assay was used to test the binding of MPS–NSSLs–SPANb with SP-A antigen. A total of 100 µL of SP-A antigen at a concentration of 5 µg/mL was added to a protein-binding plate and incubated overnight at 4 °C, followed by blocking with 0.5% albumin in Tris-buffered saline (TBS). After three washes with TBS containing 0.05% Tween 20 (TBST), 100 µL of SPANb–FITC, MPS–NSSLs–SPANb–FITC, MPS–NSSLs, and PBS was added to the plate and incubated at 4 °C for 1 h in the dark. After washing the plate three times with TBST, signals of FITC were read on a Victor^2^ 1420 multiple plate reader (Perkin Elmer, Waltham, MA).

### Characteristics of MPS–NSSLs–SPANb

#### Particle size distribution of MPS–NSSLs–SPANb

The particle size distribution of MPS–NSSLs–SPANb was measured using a Malvern Zetasizer Nano ZS (Malvern Instruments, Worcestershire, UK). Samples were diluted three times and transferred to disposable low-volume plastic cuvettes, and then analyzed at 25 °C with refractive indices of phospholipids as materials and 0.9% NaCl (w/v) as the bulk diluent used to calculate the hydrodynamic particle sizes.

#### Cryo-TEM of MPS–NSSLs–SPANb

Cryo-TEM was used to visualize the morphology of MPS–NSSLs–SPANb. Copper grids (200-mesh) coated with perforated carbon film (Lacey carbon film, ProSci Tech, Kirwan, Australia) were glow discharged in nitrogen to render them hydrophilic and placed in a laboratory-built humidity-controlled vitrification system. Aliquots of samples were applied onto the grids, and after 30 s adsorption time, grids were blotted manually by filter paper for approximately 3 s. Grids were then plunged into liquid ethane cooled by liquid nitrogen. Images were recorded using a Tecnai 12 transmission electron microscope (TEM) operating at 120 kV and equipped with an FEI Eagle 4k × 4k charge-coupled device (FEI, Eindhoven, The Netherlands). At all times, low-dose procedures were followed, limiting the electron dose to no more than 10 electrons/Å^2^.

#### Encapsulation efficiency of MPS

The encapsulation efficiency of MPS–NSSLs–SPANb to MPS was determined by a high-performance liquid chromatograph (HPLC) system (LC-20AD, Shimadzu, Kyoto, Japan). Chromatography was performed on a Inertsil C18 column (150 mm × 4.6 mm, 5 µm) utilizing a mobile phase of 0.34% potassium dihydrogen phosphate/methanol buffer (35:65) at a column temperature of 30 °C and a flow rate of 1 mL/min with UV detection at 245 nm. The loading volume was 20 µL. To plot the standard curve, MPS was precisely weighed and serially dissolved in the mobile phase to a concentration of 200, 100, 50, 25, 12.5, 6.25, 3.125, and 1.56 µg/mL, followed by HPLC detection with a loading volume of 20 µL. A linear regression equation was obtained by plotting the peak area (*Y*) versus the MPS concentration (*X*).

After drug loading, the unpurified MPS–NSSLs (0.5 mL) was first conjugated with SPANb to form MPS–MSSLs–SPANb. Then, the unpurified MPS–MSSLs–SPANb (0.5 mL) was treated with 5 mL of methanol to dissolve MPS–NSSLs–SPANb nanoparticles, and the total amount of MPS was measured using HPLC. Another 0.5 mL of MPS–MSSLs–SPANb was purified by gel filtration, and the amount of free MPS was measured by HPLC. The encapsulation efficiency of MPS was calculated using the following formula:

$$\text{EE\%=}\frac{{\text{W}}_{\text{T}}{\text{-W}}_{\text{F}}}{{\text{W}}_{\text{T}}}\text{ × 100\%}$$

where W_T_ represents the total amount of MPS in MPS–NSSLs–SPANb suspension, and W_F_ represents the amount of free MPS that was not encapsulated.

#### Retention of MPS–NSSLs–SPANb drug loading

MPS–NSSLs–SPANb suspension was divided into three aliquots and preserved at 4 °C. Samples were taken, and the encapsulation efficiency was measured at 0, 4, 8, and 12 weeks after MPS–NSSLs–SPANb preparation.

### Distribution of MPS–NSSLs–SPANb in nude mice

#### Ethics statement

This study was approved by Institutional Animal Ethics Committee for Experimentation on Animals of Tongji University.

#### Small animal imaging

As the amino acid sequences of SP-A in rats and nude mice are highly homologous (95%), and nude mice are easier for *in vivo* imaging, the present study used nude mice as experimental animals. Labeled rat SPANb with FITC (SPANb–FITC) was used to observe the *in vivo* distribution of SPANb. Nude mice were randomly assigned into five groups (*n* = 1), anesthetized, and intravenously injected with the same dose of SPANb–FITC, MPS–NSSLs–SPANb–FITC, NSSLs–SPANb, MPS–NSSLs–NBD, and NSSLs–NBD. The distribution of liposome particles in mice was observed using a small animal imaging system (NightOWL LB-983, Berthold, Bad Wildbad, Germany) at 15 min, 1 h, 3 h, 6 h, and 8 h after injection.

### Pharmacokinetic study of MPS–NSSLs–SPANb in rats

#### Animal grouping and treatment

A total of 105 healthy male Sprague Dawley (SD) rats (102 ± 8 g) were randomly assigned into three groups (*n* = 35): the MPS–NSSLs–SPANb, MPS–NSSLs, and MPS groups. Rats in the three groups were intravenously injected with MPS–NSSLs–SPANb, MPS–NSSLs, and MPS, respectively, at an MPS dose of 2 mg/kg, and sacrificed at 15 min, 30 min, 1 h, 2 h, 4 h, 8 h, and 12 h after injection.

#### Pretreatment of tissue samples and preparation of HPLC samples

At each time point, five rats from each group were taken and anesthetized by isoflurane for orbital blood sampling. Plasma was isolated by centrifuging the blood with anticoagulant EDTA. After blood sampling, animals were sacrificed and their heart, liver, spleen, lung, and kidney tissues were sampled, washed by saline, dried on a filter paper, and precisely weighed. Plasma and tissue samples were preserved at −20 °C for future use. MPS concentrations in plasma and tissues were measured by HPLC (LC-20AD, Shimadzu, Kyoto, Japan). Chromatography was performed on a Inertsil C18 column (150 mm × 4.6 mm, 5 µm) utilizing a mobile phase of 0.34% potassium dihydrogen phosphate/methanol buffer (35:65) at a column temperature of 30 °C and a flow rate of 1 mL/min with UV detection at 245 nm. The loading volume was 20 µL.

#### Evaluating the tissue-targeting property of MPS–NSSLs–SPANb

The tissue specificity of MPS–NSSLs–SPANb and MPS–NSSLs was evaluated using the peak concentration ratio (*Ce*) and the relative uptake rate (*Re*).

$$Ce=\frac{{\left(C_{p}\right)}_{a}}{{\left(C_{P}\right)}_{b}}$$

where *C_p_* represents the peak value of the drug, *a* represents the drug distribution in a specific organ after injecting MPS–NSSLs–SPAN or MPS–NSSLs, and *b* represents the drug distribution in the same organ after injecting MPS. The *Ce* value represents the difference in drug distribution in each organ or tissue; a higher *Ce* value indicates a greater difference in drug distribution.

$$Re=\frac{{\left(AUC_{i}\right)}_{a}}{{\left(AUC_{i}\right)}_{b}}$$

where *AUC_i_* represents the area under the concentration–time curve (calculated by OriginPro 9.0 using the concentration–time curve) and *i* represents a specific tissue or organ. *Re* > 1 indicates the drug targets at tissue or organ *i*; the higher the *Re* value, the better the targeting effect. *Re* < 1 indicates that the drug is nonspecific to tissue or organ *i*.

### Therapeutic effect of MPS–NSSLs–SPANb in rats with bleomycin-induced ALI

#### Animals

Animal experiments were performed using male SD SPF rats, 4–5 weeks old, weighing 90 ± 10 g and nude mice, 5–6 weeks old, weighing 15 ± 5 g. The rats/mice were purchased from the SLAC Laboratory Animal Ltd. Co. (Shanghai, China). All animal experiments were approved by the Institutional Animal Ethics Committee for Experimentation on Animals of Tongji University.

#### Animal grouping

A total of 60 male SD rats (98.8 ± 5.0 g) were randomly assigned into six groups: (1) ALI rats treated with normal dose of MPS–NSSLs–SPANb (group A, MPS dose at 1 mg/kg); (2) ALI rats treated with low dose of MPS–NSSLs–SPANb (group B, MPS dose at 0.5 mg/kg); (3) ALI rats treated with untargeted MPS–NSSLs (group C, MPS dose at 1 mg/kg); (4) ALI rats treated with MPS (group D, MPS dose at 1 mg/kg); (5) untreated ALI rats (group E); and (6) healthy control (group F). Each group was further divided into two subgroups (*n* = 5), with one subgroup sampled after 1 week and another after 2 weeks.

Lung injury was induced in the rats in groups A–E by an intratracheal injection of bleomycin at a dose of 5 mg/kg, and the rats in group F were injected with equal volumes of saline. After establishing the model, the rats in groups A–D were intravenously injected once daily, with MPS–NSSLs–SPANb (MPS dose at 1 mg/kg), MPS–NSSLs–SPANb (MPS dose at 0.5 mg/kg), MPS–NSSLs (MPS dose at 1 mg/kg), and MPS (1 mg/kg), respectively, and groups E and F received a saline injection of equal volumes.

A further 60 SD rats were grouped and followed the same treatment as described above. These were fed in a nonsterile environment. Bronchoalveolar lavage fluid (BALF) was collected and used for culturing bacteria and fungi. Lavage administration was done four times for each rat, 2.5 mL of 0.9% NaCl each time.

For evaluating animal survival, 72 SD rats (99 ± 5 g) were grouped as above (*n* = 12) and modeled (groups A–E) by an intratracheal injection of bleomycin at a dose of 10 mg/kg, and group F received a saline injection of equal volumes. Each group was intervened for 2 weeks in the same way as described above, and the rat survival of each group was observed.

#### Pathological changes of the lung tissue

The right middle lobe of rat lungs was sampled, fixed in 4% glutaraldehyde, embedded in paraffin, sectioned, stained with hematoxylin–eosin (HE), and observed under a Leica SCN400 imaging system. Biopsy was prepared and provided by WuHan Google Biotechnology Company (Shanghai, China). Based on the level of alveolar congestion, hemorrhage, inflammatory cell infiltration, alveolar structure damage, and hyaline membrane formation, the degree of severity of pathological changes in the lung tissues was evaluated according to the scoring standards (Ranieri et al., 2012) listed in Table 1.

**Table 1.** Scoring standard of pathological change in lung tissues.

Degree of injury	Score
Minimal	0
Mild	1
Moderate	2
Severe	3
Maximal	4

#### Bacterial and fungal cultures of the BALF

The left lung of rats was lavaged with saline (2.5 mL each time, four times in total) under strictly aseptic conditions; the BALF was collected and the volume was recorded. One mL of BALF was taken for bacterial and fungal culture; the remaining was centrifuged at 3000 rpm for 15 min, and the supernatant was preserved at –80 °C for further treatment.

#### Content of TNF-α, IL-8, and TGF-β1 in BALF

The concentration of TNF-α, IL-8, and TGF-β1 in BALF was detected by the avidin–biotin complex enzyme-linked immunosorbent assay (ABC-ELISA).

#### Expression of NF-κB mRNA in lung tissue

The right upper lobe of rat lungs was sampled and preserved at –80 °C, and the expression of NF-κB mRNA was detected using reverse transcription polymerase chain reaction (RT-PCR).

#### Verifying the safety of MPS–NSSLs–SPANb

The orbital blood of rats was sampled and serum isolated to measure the level of alanine aminotransferase (ALT), aspartate aminotransferase (AST), urea nitrogen (BUN), creatinine (Cr), and other indicators using an automatic biochemical analyzer, thereby evaluating the function of rat liver and kidney.

### Statistical analysis

The statistical analysis was performed using the software SPSS 20.0 (SPSS Inc., Chicago, IL). Continuous variables were presented as mean ± standard deviation. Multi-group difference was compared by analysis of variance; intergroup comparison was performed by *t*-test; sample rate and grading data (such as semi-quantitative scoring) were compared by *χ*^2^-test; intergroup survival curve comparison was performed by log rank test. Statistical significance was defined at *p* < .05.

## Results

### Establishment of MPS-NSSLs-SPANb nanoparticles

**Figure 1.**
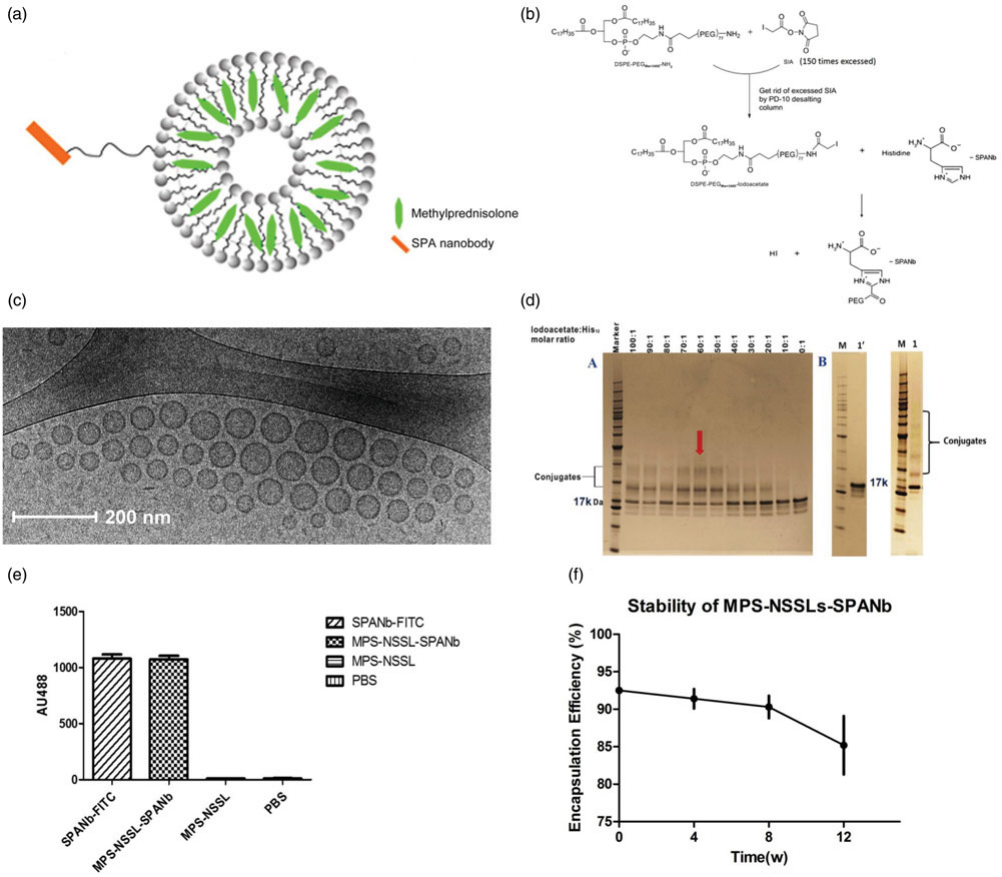
(a) Schematic of MPS-NSSLs-SPANb nanoparticles. (b) The conjugation was achieved by the reaction between the iodoacetyl group from liposomes and the both imidazolyl side chain nitrogens of histidine functional groups within nanobody. (c) Cryo-TEM images of extruded actively targeted liposomal MPS-NSSLs-SPANb nanoparticles. (d) SDS-PAGE of conjugating SP-A nanobody to liposomes, at a iodoacetate-to-His12 ratio 60:1. 1′ represents SP-A nanobody, 1 represents unpurified MPS-NSSLs-SPANb. (e) ELISA test of SP-A nanobody and different nanoparticles to SP-A antigen. All nanoparticles were purified by gel-filtration to get rid of free nanobody, and fluorescence was quantified at the same level before ELISA test. (f) MPS–NSSLs–SPANb was stored at 4 °C, encapsulation efficiency of MPS–NSSLs–SPANb to MPS was tested every 4 weeks.

The present study has coupled the SPANb to MPS loaded liposomes. Chemical functionality was added to the liposomes. The nanobody was conjugated to a nano-sterically stabilized liposome (NSSL) into which MPS was loaded. The liposomes consisted of 1,2-distearoyl-sn-glycero-3-phosphocholine (DSPC), CHOL, and DSPE-PEG3400-iodoacetate. The vesicles were loaded with MPS using a calcium gradient approach prior to conjugation with the nanobody (Wyss et al., 2009). The DSPE-PEG3400-iodoacetate moiety provided functionality for conjugation to the His-Tag present on the nanobody as well a stealth corona to promote enhanced *in vivo* circulation (Figure 1(a)). The conjugation was achieved by the reaction between the iodoacetyl group from liposomes and the imidazolyl side chain nitrogens of histidine functional groups within nanobody. The extent of conjugation is observable in Figure 1(b). The morphology of the particles post-preparation was examined to ensure that they were fit-for-purpose. Dynamic light scattering showed that the average particle size of MPS–NSSLs–SPANb was 106 nm in diameter in average (polydispersity index: 0.263). The unilamellar round morphology of the nanoparticles was confirmed by cryo-TEM. The nanoparticles were well-dispersed with no aggregation (Figure 1(c)). SDS-PAGE showed new bands after incubating DSPE-PEG3400-iodoacetate with SPANb, indicating successful conjugation between iodoacetate moiety and the His-tag on SP-A. The mass increase was around 3.4 kDa, correlating to the addition of DSPE-PEG3400-iodoacetate per SPANb. The image analysis demonstrated that the optimal molar ratio of DSPE-PEG3400-iodoacetate:SPANb was 60:1. To prepare the actively targeted nanoparticles, the drug loaded liposomes and SPANb were incubated (60:1 ratio of DSPE-PEG3400-iodoacetate:SPANb) to form MPS–NSSLs–SPANb. SDS-PAGE (Figure 1(d)) as confirmed by SDS-PAGE. Image analysis determined that at a molar ratio of DSPE-PEG3400-iodoacetate:SPANb of 60:1, the reaction efficiency between SPANb and liposomes was 36 ± 2%. ELISA test (Figure 1(e)) identified that there was no significant difference (*p* > .05) between MPS–NSSLs–SPANb and SPANb in antigen-binding capability, indicating retention of specificity of the nanobody following conjugation. The untargeted MPS–NSSLs and the PBS buffer control showed little interaction with the SP-A antigen as expected.

Removal of free MPS was performed using gel filtration on a Superose 12 10/300 column, the encapsulation efficiency of MPS–NSSLs–SPANb to MPS reached 92.5 ± 0.5%, and the ratio drug:lipid was approximately 0.40. After 12 weeks of storage at 4 °C, the encapsulation efficiency was not significantly altered (*p* > .05), indicating good stability of the samples (Figure 1(f)).

### Small animal imaging of MPS–NSSLs–SPANb *in vivo*

**Figure 2.**
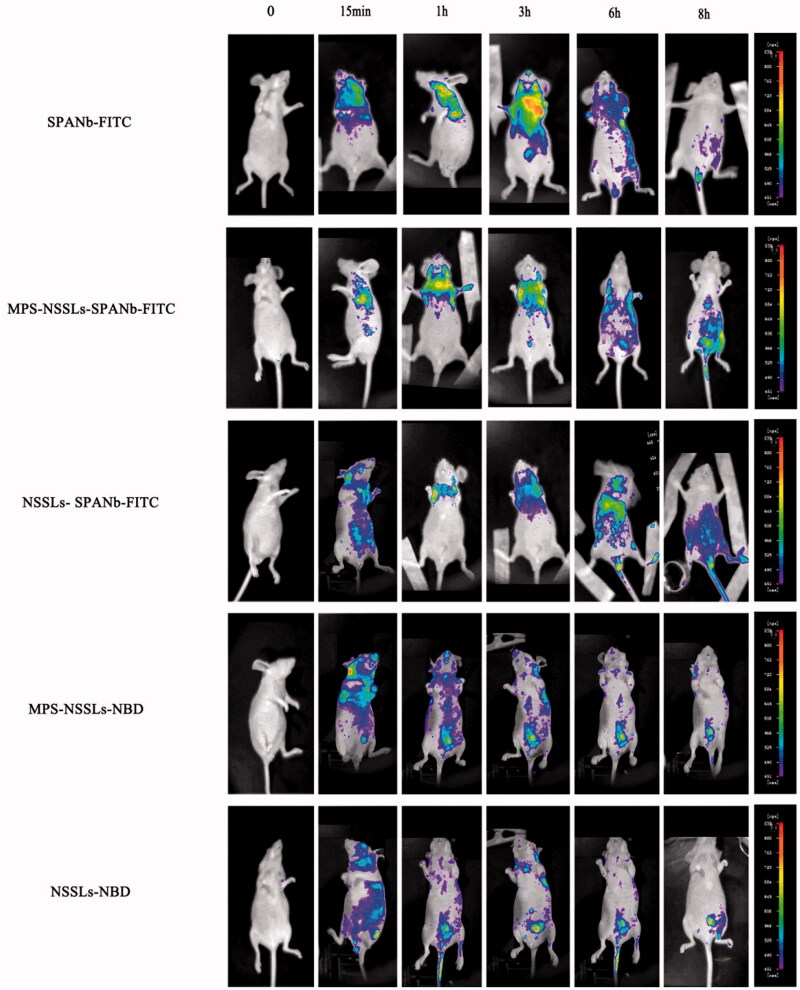
Lung-targeting analysis of MPS-NSSLs-SPANb in nude mice by small animal imaging. The experiments were performed independently three times, and showed similar results.

The active targeting nature of the liposome was assessed *in vivo*. Although the liposomes used in this study were functionalized with rat SPANb, nude mice were chosen as experimental animals to determine biodistribution because nude mice were more suitable for *in vivo* imaging and the amino acid sequence of SP-A of nude mice is highly homologous (95%) to that of rat. As shown in Figure 2, a significant lung accumulation of nanoparticles was observed at 15 min after the injection of FITC-labeled MPS–NSSLs–SPANb and NSSLs–SPANb, and even at 6 h after injection, drug uptake could still be observed at the lung tissues. No significant liposome aggregation was observed at other organs, and the metabolism of liposomes before and after drug loading exhibited no significant differences. Particles with no active targeting showed little accumulation in lung tissue.

### Distribution in plasma and other tissues of MPS, MPS-NSSLs, and MPS-NSSLs-SPANb

**Figure 3.**
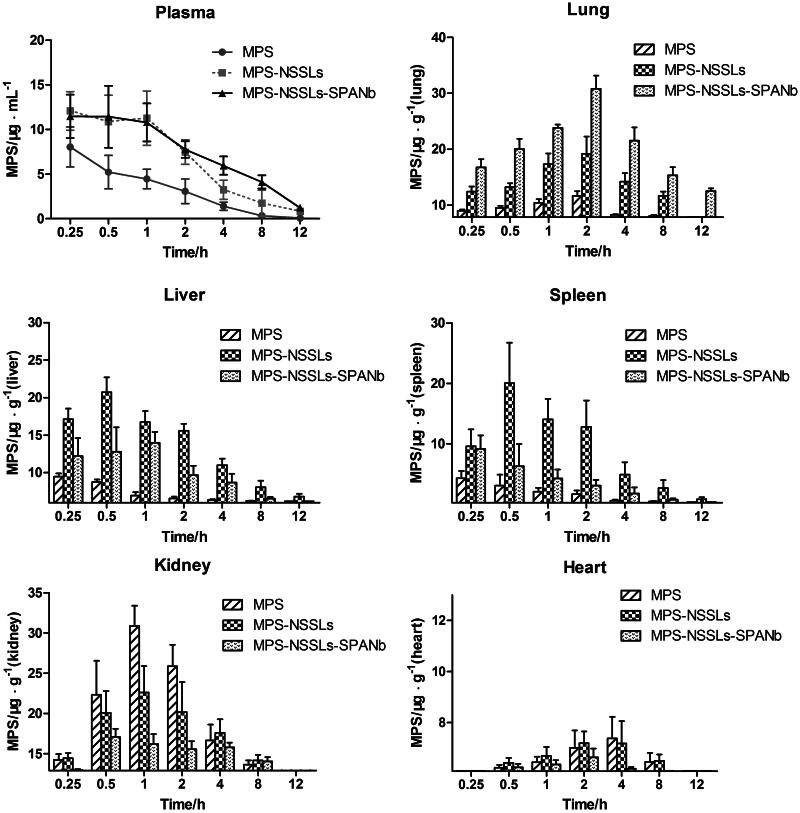
MPS distribution in plasma and other tissues after injection of MPS, MPS-NSSLs and MPS-NSSLs-SPANb. MPS-NSSLs-SPANb lengthened methyprednisolone’s blood circulation time and increased MPS concentration in both blood and lung tissue.

Pharmacokinetic studies were performed in SD rats to assess the circulation time of the nanoparticles. As shown in Figure 3, actively targeted MPS–NSSLs–SPANb lengthened MPS blood circulation time and increased MPS concentration in the blood. At each time point after injection, the MPS concentration in blood and lungs in rats injected with MPS–NSSLs–SPANb significantly exceeded that of rats injected with MPS (*p* < .05 and *p* < .01, respectively). The peak MPS concentration measured in the lung tissue, two hours after injection, with the lung targeted liposomes (MPS–NSSLs–SPANb) (30.8 ± 5.2 µg/g) was 1.5 times of that of the untargeted liposome species (MPS–NSSLs) (19.2 ± 6.9 µg/g). Even 12 hours after the injection, the peak MPS concentration in lungs of MPS–NSSLs–SPANb group (12.5 ± 1.1 µg/g) was still higher than that of MPS-NSSLs group (7.83 ± 0.01 µg/g). Both the MPS–NSSLs–SPANb and MPS–NSSLs groups showed an increasing amount of MPS in the liver and spleen, but this increase was more significant in the MPS-NSSLs group.

### Evaluating the tissue-targeting properties of MPS–NSSLs and MPS–NSSLs–SPANb

To assess whether the conjugated nanobodies can target the lung, the levels of MPS in the lungs for different experimental groups were assessed. As shown in Figure S1, MPS–NSSLs–SPANb exhibited remarkable lung-targeting properties compared to the untargeted nanoparticles. The peak concentration value of MPS in lungs after the injection of the targeted MPS–NSSLs–SPANb particles was 4.58-fold (*Ce* = 4.58) compared to the MPS injection and 1.22 fold compared to the MPS–NSSLs injection. The AUC at 12 h after the MPS–NSSLs–SPANb injection (*AUC*_0–12 h_) was 17.78-fold (*Re* = 17.78) of that after the MPS injection and 1.47-fold the MPS–NSSLs. Due to phagocytosis, MPS–NSSLs and MPS–NSSLs–SPANb were also enriched in the liver and spleen, but the accumulation of MPS in the liver and spleen was lower in the MPS–NSSLs–SPANb group than in the MPS–NSSLs group. The *Re* and *Ce* values of MPS–NSSLs in the heart and kidney were both  < 1, indicating that these organs were not targeted by MPS–NSSLs.

### Therapeutic effect of MPS–NSSLs–SPANb on rat lung injury

**Figure 4.**
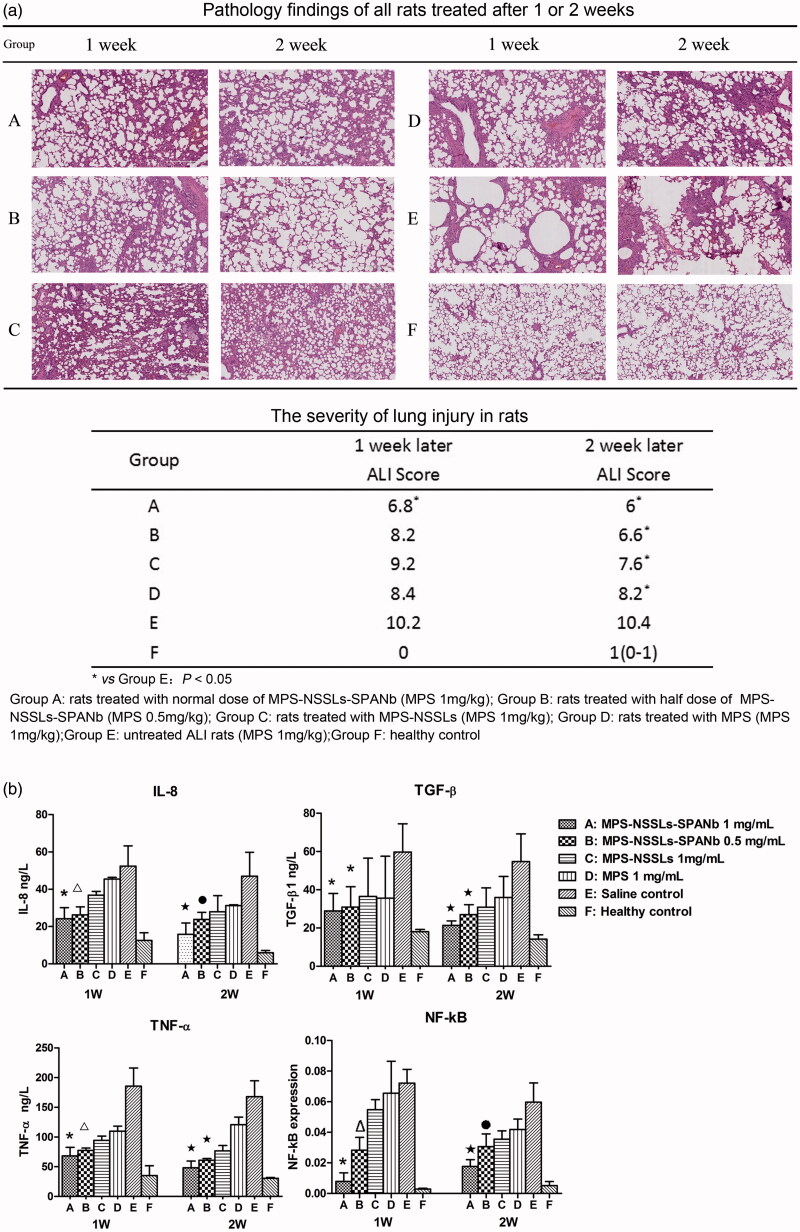
Therapeutic effect of nanoparticles. (a) Lung injury on rats model was highly pathologically improved after treating with MPS-NSSLs-SPANb 1 and 2 weeks later. (b) MPS-NSSLs-SPANb significantly reduced the expression of inflammation cytokine in both BALF and lung tissue.

Acute lung injury was induced in SD rats by intratracheal injection of bleomycin at a dose of 5 mg/kg (groups A–E). Group F was a saline control. For each treatment group, two time points were set-up with *n* = 5 per group and per time point. The extent of lung injury and treatment was assessed using histopathological HE staining of the right middle lobe of the rat lungs. The HE staining of pathological lung samples (Supplementary Information) showed that after 1 week of bleomycin modeling, group E (untreated) developed a significant pulmonary inflammation, with alveolar wall thickening, alveolar structural damage, extensive inflammatory cell infiltration, interval widening, and massive pulmonary consolidation. Such inflammation was alleviated after MPS treatment, but an improvement in lung injury was more significant in rats injected with MPS–NSSLs–SPANb (groups A and B). The degree of severity of lung damage was scored by the method shown in supplementary and the results (pathology findings and scoring) are shown in Figure 4(a). As indicated, after 1 week of treatment, the rats injected with a normal dose of MPS–NSSLs–SPANb (group A) showed a significantly alleviated inflammation compared with group E (*p* < .05), but those injected with a lower dose of MPS–NSSLs–SPANb (group B), MPS–NSSLs (group C), and MPS (group D) exhibited an insignificant improvement in inflammation compared with group E (*p* > .05). After 2 weeks of treatment, lung injury was still severe in rats without intervention (group E), but it significantly attenuated (*p* < .05) in rats receiving treatment (groups A–D) compared with group E. Notably, rats in group D (MPS) showed only a little improvement in the second week of treatment compared with the previous week, yet inflammation in group B (low-dose MPS–NSSLs–SPANb) and C (MPS–NSSLs) continued to alleviate in the following week of intervention.

The bacterial and fungal levels in BALF were assessed. The culturing of microorganisms detected no bacterial or fungal growth in the BALF of rats receiving the treatment of MPS–NSSLs–SPANb at full or half dose, indicating that MPS–NSSLs–SPANb did not increase the chances of infection during the treatment in treated animals (Table 2). TNF-α, IL-8, and TGF-β1 levels in BALF were quantified to assess the levels of the cytokines following administration. Expression of NF-κB mRNA in lung tissues was also assessed. As shown in Figure 4(b), our targeted liposomes (MPS–NSSLs–SPANb) could significantly reduce the expression of inflammatory cytokines in both BALF and lung tissue (*p* < .05).

**Table 2.** Culturing bronchoalveolar lavage fluid to test bacterial and fungal infection.

Group	1 week later	2 weeks later
A: MPS-NSSSLs-SPANb 1 mg/mL	–	–
B: MPS-NSSSLs-SPANb 0.5 mg/mL	–	–
C: MPS-NSSLs 1 mg/mL	3 cases: *Staphylococcus epidermidis*	1 case: *Staphylococcus epidermidis* 1 case: *Escherichia Coli*
D: MPS 1 mg/mL	–	1 case: *Staphylococcus epidermidis* 1 case: *Proteus penneri*
E: Saline control	–	–
F: Healthy control	–	–

After lung injury was induced with 10 mg/kg of bleomycin, rats were treated for 2 weeks and observed for 10 weeks. The survival curves and the survival rates of rats in each group are shown in Figure S2. At 10 weeks after the interventions, the survival rate of rats in group A (high dose MPS-NSSLs-SPANb) significantly exceeded that in group E (*p* < .05). The survival rates were insignificantly higher in groups B, C, and D than in group E (*p* > .05). Pairwise comparisons of rat survival between groups A, B, C, and D detected no significant difference.

### Verifying the safety of MPS–NSSLs–SPANb

The results of ALT, AST, BUN, and Cr detection in rat serum are shown in Table 3. As indicated, serum levels of ALT, AST, BUN, and Cr in rats from groups A and B were insignificantly different from the levels in control after 1 and 2 weeks of intervention (*p* > .05). Intergroup comparisons detected no significant difference in serum levels of ALT, AST, BUN, and Cr. Untreated rats (group E) displayed significantly higher (*p* < .05) serum levels of ALT and BUN at 2 weeks after bleomycin modeling compared with the healthy control (group F), while rats treated with MPS (group D) exhibited significantly higher (*p* < .05) ALT at 1 week after intervention and significantly higher (*p* < .05) Cr at 2 weeks after the intervention compared with the healthy control (group F). These results indicated that MPS–NSSLs–SPANb was highly safe for short-term treatment.

**Table 3.** Serum levels of ALT, AST, BUN and Cr in different groups of rats.

Group	ALT (IU/L)	AST (IU/L)	BUN (mmol/L)	Cr (μmol/L)
A				
1w	46.75 ± 5.56	93.33 ± 12.34	7.5 ± 1.058	11 ± 2.16
2w	51.86 ± 26.74	94.43 ± 34.22	6.143 ± 1.685	13.75 ± 11.83
B				
1w	53.4 ± 9.659	91.25 ± 15.86	6.42 ± 0.8136	14.4 ± 6.656
2w	95.8 ± 63.69	111.5 ± 54.49	6.8 ± 1.532	11.83 ± 1.722
C				
1w	70.33 ± 53.17	143.2 ± 124.3	4.95 ± 0.7688	10.67 ± 1.633
2w	64.8 ± 28.15	143.7 ± 79.72	6.271 ± 0.9447	15.33 ± 1.528
D				
1w	58.4 ± 7.436*	121.4 ± 52.67	6.62 ± 0.795	10.8 ± 2.387
2w	38.17 ± 6.242	66 ± 12.35	6.633 ± 0.8595	13.67 ± 2.422*
E				
1w	47.33 ± 6.088	85.83 ± 12.7	5.983 ± 2.369	12.33 ± 2.658
2w	53.33 ± 24.23	95.83 ± 60.03	7.333 ± 1.621*	13.17 ± 6.706
F				
1w	44 ± 4.583	75.4 ± 3.912	5.667 ± 0.2082	11.6 ± 1.517
2w	34.4 ± 5.32	92.6 ± 16.53	5.18 ± 0.8871	9 ± 2.449

* vs. group F *p* < .05.

## Discussion

The development of lung-targeted drug delivery mainly depends on the specific molecules conjugated with the drug-loaded liposomes. The SP-A is highly specific for lungs, as it shows the most abundant expression in lung tissues while extremely low levels in extra-pulmonary tissues. Thus, it is considered an ideal molecule for lung targeting. Our previous study used SP-A polyclonal antibody (PcAb) as lung-specific targeting molecules to prepare SP-A PcAb-conjugated and dexamethasone-loaded liposomes, and carried out animal experiments to confirm the feasibility of using SP-A as a lung-specific targeting molecule (Chen et al., 2013). Recently, anti-rat SP-A nanobodies (rSPANb) were developed by our research group and we have shown that anti-rat SP-A nanobodies are highly lung-specific and have a good safety profile (Wang et al., 2015).

Using SPANb as a lung-targeting agent overcame the defects of the whole SP-A antibody, such as high molecular weight, high liver/spleen phagocytosis, and strong immunogenicity. Using NSSLs as a drug carrier and preparing liposomes with film dispersion guaranteed the uniform size and distribution of particles, and hydrophilic modification of the particles with PEG reduced cell phagocytosis and increased the dimensional stability of particles. Using pH gradient to actively load MPS into liposomes achieved the highest loading capacity as well as the ratio between the drug and the lipids. Thus, the new lung-targeting GC drug MPS–NSSLs–SPANb demonstrated an encapsulation efficiency as high as more than 90%, and could be stably stored at 4 °C for no less than 12 weeks.

To detect the distribution of this new lung-targeting drug, nude mice were used for *in vivo* imaging because animal hairs would affect florescence observation and the amino acid sequence of rat SP-A was highly homologous (95%) to that of mice. At 15 min after injection of FITC-labeled MPS–NSSLs–SPANb and NSSLs–SPANb, significant lung accumulation was observed and, even at 6 h after injection, drug uptake was still observed at lung tissues yet not in other organs. However, liposomes (with or without drug carrying) without SPANb conjugation all showed systemic (nonspecific) distribution at 1 h after injection, indicating that SPANb-conjugated MPS–NSSLs was highly specific and lengthened the stay of drugs in the lung tissues. Pharmacokinetic studies showed that the MPS concentration in the blood of MPS–NSSLs–SPANb group was significantly higher than that in MPS group at all of the time points. The peak MPS concentration of MPS–NSSLs–SPANb group in lung tissues was 4.58-fold of that in MPS group, and the 12-h AUC (AUC_0–12 h_) of MPS–NSSLs–SPANb group was 17.78-fold of that in MPS group. The new drug in our present study improved the accumulation of MPS at lung tissues and increased the blood circulation time. Thus, it provided the basis for the targeted treatment of lung injury in the future.

Animal experiments showed that MPS–NSSLs–SPANb was highly effective and less toxic when used for lung injury treatment. The rats treated with MPS–NSSLs–SPANb (1 mg/kg/d) showed significantly alleviated pathological changes after 1–2 weeks of the intervention and a higher survival rate than untreated rats. The rats treated with MPS–NSSLs–SPANb at half dose also displayed outcomes comparable with those treated with full dose. The attenuation in lung injury with the use of this drug might be attributed to the anti-inflammatory effects of corticosteroids (Mikawa et al., 2003).

MPS–NSSLs–SPANb significantly decreased the levels of TNF-α, IL-8, and TGF-β1 in rat BALF and NF-κB in the lung tissue. The culturing of microorganisms detected no bacterial or fungal growth in the BALF of rats receiving the treatment of MPS–NSSLs–SPANb at full or half dose, indicating that MPS–NSSLs–SPANb did not increase the chances of infection during the treatment. No significant damage to liver or kidney was observed after 1–2 weeks of intervention, indicating that MPS–NSSLs–SPANb is highly safe for short-term treatment.

## Conclusions

This study for the first time developed a lung-targeting GC (MPS–NSSLs–SPANb) using SPANb as the lung-targeting molecule. This drug was highly specific for lung tissues, and was more effective and less toxic in treating rats with bleomycin-induced lung injury.

## References

[CIT0001] Angus DC. (2012). The acute respiratory distress syndrome: what’s in a name? JAMA 307:2542–4. 22797455 10.1001/jama.2012.6761

[CIT0002] Ashbaugh DG, Bigelow DB, Petty TL, et al. (2005). Acute respiratory distress in adults. The Lancet, Saturday 12 August 1967. Crit Care Resusc 7:60–1. 16548822

[CIT0003] Bernard GR, Artigas A, Brigham KL, et al. (1994). The American-European Consensus Conference on ARDS. Definitions, mechanisms, relevant outcomes, and clinical trial coordination. Am J Respir Crit Care Med 149:818–24. 7509706 10.1164/ajrccm.149.3.7509706

[CIT0004] Buregeya E, Fowler RA, Talmor DS, et al. (2014). Acute respiratory distress syndrome in the global context. Glob Heart 9:289–95. 25667180 10.1016/j.gheart.2014.08.003

[CIT0005] Chen XY, Wang SM, Li N, et al. (2013). Creation of lung-targeted dexamethasone immunoliposome and its therapeutic effect on bleomycin-induced lung injury in rats. PLoS One 8:e58275. 23516459 10.1371/journal.pone.0058275 PMC3597622

[CIT0006] Courrier HM, Butz N, Vandamme TF. (2002). Pulmonary drug delivery systems: recent developments and prospects. Crit Rev Ther Drug Carrier Syst 19:425–98. 12661699 10.1615/critrevtherdrugcarriersyst.v19.i45.40

[CIT0007] Curtis JR, Westfall AO, Allison J, et al. (2006). Population-based assessment of adverse events associated with long-term glucocorticoid use. Arthritis Rheum 55:420–6. 16739208 10.1002/art.21984

[CIT0008] Dandekar P, Venkataraman C, Mehra A. (2010). Pulmonary targeting of nanoparticle drug matrices. J Aerosol Med Pulmon Drug Deliv 23:343–53. 10.1089/jamp.2009.0784 20455773

[CIT0009] Desmyter A, Spinelli S, Roussel A, Cambillau C. (2015). Camelid nanobodies: killing two birds with one stone. Curr Opin Struct Biol 32:1–8. 25614146 10.1016/j.sbi.2015.01.001

[CIT0010] Ferguson ND, Kacmarek RM, Chiche JD, et al. (2004). Screening of ARDS patients using standardized ventilator settings: influence on enrollment in a clinical trial. Intensive Care Med 30:1111–16. 14991096 10.1007/s00134-004-2163-2

[CIT0011] Gajic O, Dabbagh O, Park PK, et al. (2011). Early identification of patients at risk of acute lung injury: evaluation of lung injury prediction score. Am J Respir Crit Care Med 183:462–70. 20802164 10.1164/rccm.201004-0549OC PMC3056224

[CIT0012] Greenstein S, Ghias K, Krett NL, Rosen ST. (2002). Mechanisms of glucocorticoid-mediated apoptosis in hematological malignancies. Clin Cancer Res 8:1681–94. 12060604

[CIT0013] Hamers-Casterman C, Atarhouch T, Muyldermans S, et al. (1993). Naturally occurring antibodies devoid of light chains. Nature 363:446–8. 8502296 10.1038/363446a0

[CIT0014] Irwin RS, Richardson ND. (2006). Side effects with inhaled corticosteroids: the physician’s perception. Chest 130:41S–53S. 16840367 10.1378/chest.130.1_suppl.41S

[CIT0015] Maybauer MO, Maybauer DM, Herndon DN. (2006). Incidence and outcomes of acute lung injury. N Engl J Med 354:416–17. 16444810

[CIT0016] Mikawa K, Nishina K, Takao Y, Obara H. (2003). ONO-1714, a nitric oxide synthase inhibitor, attenuates endotoxin-induced acute lung injury in rabbits. Anesth Analg 97:1751–5. 14633554 10.1213/01.ANE.0000086896.90343.13

[CIT0017] Pan D, Lanza GM, Wickline SA, Caruthers SD. (2009). Nanomedicine: perspective and promises with ligand-directed molecular imaging. Eur J Radiol 70:274–85. 19268515 10.1016/j.ejrad.2009.01.042

[CIT0018] Ranieri VM, Rubenfeld GD, Thompson BT, et al. (2012). Acute respiratory distress syndrome: the Berlin Definition. JAMA 307:2526–33. 22797452 10.1001/jama.2012.5669

[CIT0019] Rochat TS, Janssens JP. (2012). Systemic and oropharyngeal side effects of inhaled corticosteroids. Rev Med Suisse 8:2219–23. 23240297

[CIT0020] Schmiedl A, Ochs M, Muhlfeld C, et al. (2005). Distribution of surfactant proteins in type II pneumocytes of newborn, 14-day old, and adult rats: an immunoelectron microscopic and stereological study. Histochem Cell Biol 124:465–76. 16187065 10.1007/s00418-005-0066-0

[CIT0021] Siontorou CG. (2013). Nanobodies as novel agents for disease diagnosis and therapy. Int J Nanomedicine 8:4215–27. 24204148 10.2147/IJN.S39428 PMC3818023

[CIT0022] Thompson BT, Matthay MA. (2013). The Berlin definition of ARDS versus pathological evidence of diffuse alveolar damage. Am J Respir Crit Care Med 187:675–7. 23540876 10.1164/rccm.201302-0385ed

[CIT0023] Wang SM, He X, Li N, et al. (2015). A novel nanobody specific for respiratory surfactant protein A has potential for lung targeting. Int J Nanomedicine 10:2857–69. 25926731 10.2147/IJN.S77268 PMC4403696

[CIT0024] Warrington TP, Bostwick JM. (2006). Psychiatric adverse effects of corticosteroids. Mayo Clin Proc 81:1361–7. 17036562 10.4065/81.10.1361

[CIT0025] Wyss C, Schaefer SC, Juillerat-Jeanneret L, et al. (2009). Molecular imaging by micro-CT: specific E-selectin imaging. Eur Radiol 19:2487–94. 19440717 10.1007/s00330-009-1434-2

[CIT0026] Zhai J, Scoble JA, Li N, et al. (2015). Epidermal growth factor receptor-targeted lipid nanoparticles retain self-assembled nanostructures and provide high specificity. Nanoscale 7:2905–13. 25516406 10.1039/c4nr05200e

